# At the limits of digital education. The importance of practical education for clinical competencies learning in the field of emergency medicine: A controlled non-randomized interventional study

**DOI:** 10.3389/fmed.2022.993337

**Published:** 2022-09-16

**Authors:** Lina Vogt, Michael Schauwinhold, Rolf Rossaint, Henning Schenkat, Martin Klasen, Saša Sopka

**Affiliations:** ^1^Clinic for Anesthesiology, University Hospital Rheinisch-Westfälische Technische Hochschule Aachen, Medical Faculty, Rheinisch-Westfälische Technische Hochschule Aachen, Aachen, Germany; ^2^AIXTRA – Competence Center for Training and Patient Safety, Medical Faculty, Rheinisch-Westfälische Technische Hochschule Aachen, Aachen, Germany; ^3^Dean of Students Office, Medical Faculty, Rheinisch-Westfälische Technische Hochschule Aachen, Aachen, Germany

**Keywords:** medical education, emergency medicine (MeSH database), clinical competency, practical skill, competency-based medical education (CBME), digital education

## Abstract

**Introduction:**

A high-quality education of future physicians is essential. Modern approaches interlock the acquisition of theoretical knowledge and practical skills in a spiral curriculum, leading to a mutual learning benefit for knowledge and application. This model was challenged by the elimination of hands-on trainings during the pandemic, which were often replaced by purely digital teaching models. Given the holistic nature of the spiral curriculum, we assumed that a purely digital model would have an impact on knowledge acquisition due to missing hands-on learning opportunities. The aim of the study was to investigate, using an emergency seminar as an example, whether purely digital training leads to a difference in theoretical knowledge compared to the traditional model.

**Materials and methods:**

Study design: We used a two-groups design comparing a sample of medical students taught in 2020 with a purely digital teaching format (DF; *n* = 152) with a historical control group taught with a traditional format (TF; *n* = 1060). Subject of investigation was a seminar on emergency medicine, taking place in the 4th year. Outcome parameters: The primary outcome parameter was the students' acquired knowledge, measured by the score achieved in the final exams. Students' evaluation of the seminar was used as a secondary outcome parameter.

**Results:**

Students in the DF group scored significantly lower than students in the TF group in the final exams. Students in the DF group rated the course significantly worse than students in the TF group.

**Discussion:**

The study results illustrate that purely digital education leads to inferior knowledge acquisition compared to the traditional spiral curriculum. A possible explanation may lie in a deeper processing of the information (e.g., understanding the information by experience and analysis) and accordingly a better memory recall. Moreover, the students' critical appraisal of the DF may have had an unfavorable effect on learning performance. Moderating factors may be lower learning motivation or the “zoom fatigue” effect.

**Conclusion:**

These study results clearly illustrate the importance of hands-on teaching for knowledge acquisition. The interlocking of theoretical knowledge and practical skills, as ensured by the spiral curriculum, is essential.

## Introduction

A high-quality education for future physicians is essential for each healthcare system (1–4). Modern approaches are based on the hierarchical models of Bloom's taxonomy (5) and Miller's pyramid (6) and implement them in form of a spiral curriculum (7–9). A characteristic feature of the latter is re-iterating the topics on ascending levels, while alternating theoretical lessons and practical application. This holistic approach enhances attitudes, cognitions, and skills of the students by continuously deepening the understanding of the covered topics (10). A characteristic feature of this approach is the close entanglement and temporal proximity of related theoretical and practical elements, which promotes both cognitive learning processes and skills development.

The COVID-19 pandemic with its sudden consequences for on-site teaching, however, has challenged this model (11–17). Under these conditions, medical education has changed fundamentally: large parts of the medical curricula, practical teaching modules and bedside teaching have been transferred to digital settings or virtual simulation (VS). Several previous studies have underlined the beneficial effects of simulation-based learning (SBL) and show that SBL is helpful in integrating theoretical knowledge and practice, with experience leading to better internalization of knowledge (18). Furthermore, medical SBL is effective for the acquisition of clinical skills (19, 20). Related to the field of emergency medicine SBL has long been considered as a cornerstone of training (21). Several recently published studies showed that the embedding of virtual reality (VR) in emergency medicine trainings provided auspicious zero-risk training for students (18, 21–23).

During the pandemic, digital skills education is comprehensible—and perhaps the only feasible way to acquire the respective competencies. However, the difference to hands-on training is fundamental, and the question arises whether a purely digital education can successfully maintain the spiral curriculum—or whether it leads to inferior learning outcomes.

The aim of the present study was to investigate whether a purely digital education had an influence on theoretical knowledge in the field of emergency medicine as compared to the traditional approach. Although we had reason to believe that a possible influence may be a negative one, we decided for two undirected hypotheses and postulated a difference (in any direction) between the approaches. We addressed this question on the example of an emergency medicine seminar with theoretical and practical elements. Specifically, we were interested whether a purely digital seminar led to inferior outcomes in terms of achieved points in the corresponding written exam compared to the traditional format with hands-on practical teaching. Moreover, we were interested in the subjective evaluation of the digital teaching format by the students.

## Materials and methods

### Ethics approval

This study was performed in line with the principles of the Declaration of Helsinki. The study protocol was approved by the Ethical Committee of the Faculty of Medicine of RWTH Aachen University (Chairperson Prof. Dr. med. G. Schmalzing) (EK 215-20) on June 6th, 2020. Furthermore, the study was planned, conducted, and reported according to the SQUIRE EDU Guidelines (24).

### Study design

The present study used a two-groups design comparing a novel digital teaching format (DF) with a historical control group taught by a traditional face-to-face on-site format (traditional format, TF) The study compared two teaching-learning formats in the field of emergency medicine.

During the internship in the 4th year of medical school, students learn the contents of emergency medicine, such as the organization of emergency medical services. Additional content ranges from basic life support and advanced life support to ABCDE algorithms, pediatric emergencies, and case scenarios. Furthermore, the leading symptoms of chest pain, dyspnea and loss of consciousness are subject. The covered topics, the learning content, and the learning goals were identical in both formats; they only differed with respect to the teaching modality (please see description below).

Data for DF was collected in the summer term of 2020. Since TF was not possible at the time of data collection due to COVID-19 related restrictions, we decided for a historical control. For TF, we therefore used previously acquired data sets from summer term 2015 to summer term 2019. The large data base (9 cohorts) for the historical control was chosen for two reasons. First, including data from several cohorts minimized any possible influence of unsystematic data fluctuations in the results between cohorts; second, a large control sample increases statistical power.

#### Teaching formats

In both teaching formats, the emergency medicine contents were taught on 8 days within 2 weeks. Likewise, they were identical with respect to teaching content and learning objectives.

##### Digital format

The theoretical seminars comprised a total duration of 17.75 h. These were supplemented by practical courses and sessions on self-directed learning with a total duration of 19.25 h. All group lessons were realized with the video conferencing software Zoom (Zoom Video Communications Inc., San Jose, California, USA). Practical courses used the browser-based version of the BodyInteract^TM^ software to take a medical history, carry out the initial assessment and initiate the necessary diagnostics and therapy. The cases were managed by small teams of usually three students, with one student taking over the operation of the software and the others contributing their knowledge as in a real team.

##### Traditional format

The theoretical seminars comprised a total duration of 20.25 h in the traditional format. These were supplemented by practical courses with a total duration of 22.75 h. The seminars were implemented in groups of up to 12 students in the form of an interactive, media-supported lecture. The theoretical processing of case studies was a frequently used tool. Practical exercises took place in groups of maximum 6 students. In addition to learning how to handle rescue service materials, emergency medical scenarios from the domains of resuscitation, internal medicine and traumatology were performed.

The difference in the total duration by the theoretical units was due to the omission of room switching times, breakout rooms, and the shorter time required for a single session because of the digital format.

The two formats had identical learning content and learning objectives and differed only with respect to their teaching method.

### Outcome parameters

#### Primary

The study's primary outcome parameter was the students' acquired knowledge. This was measured by the score achieved in the final examination at the end of the course in emergency medicine. The examination questions of both formats (DF and TF) were randomly selected from the same item pool; the number of examination questions as well as the maximum achievable score (20 points) were identical. For all included data sets, the examination consisted of single-choice questions with predefined correct answers; thus, there was no bias with respect to the assessment of expertise. Our analyses refer only to the emergency medicine section.

#### Secondary

Following the course, the participants rated it with a school grade according to the German system (1 = very good to 6 = insufficient). This grading was used as a secondary outcome parameter. All evaluation data in all semesters were collected with the software *Evaluna* (https://medicampus.uni-muenster.de/evaluna0.html) in digital form. The evaluation “school grade” was the final item of a questionnaire for course evaluation. Precisely, the item was “Your overall school grade for the entire course; Scale: 1 (very good) - 6 (unsatisfactory) (German: Ihre Gesamtnote für die Gesamtveranstaltung; Skala: 1 (sehr gut) - 6 (ungenügend)).

### Research hypotheses

We defined the following research hypotheses (RH) for the present study:

#### RH1 (Primary)

DF leads to a different degree of expertise than TF, as expressed in significantly different scores achieved in the final examination in the field of emergency medicine.

#### RH2 (Secondary)

DF leads to different student ratings than TF, as expressed in a significantly different school grade.

### Sample size planning

Sample size was based on the primary outcome parameter and calculated with G^*^Power 3.1.9.7 (25). Since we assumed a violation of normal distribution based on the final examination score data from previous semesters [e.g., for summer term 2019: W(128) = 0.87, *p* < 0.001, Shapiro-Wilk test], we decided to plan for a non-parametric test (Mann-Whitney U for two independent samples). Assuming a medium effect size of Cohen's *d* = 0.5, an α error probability of 0.05, a power of 0.95, and an anticipated allocation ratio of 1/9 (1 semester in the intervention group and 9 semesters in the historical control), we required a total sample size of *N* = 484, with *n* = 48 in the intervention group and *n* = 436 in the control group.

### Statistical analysis

Data were analyzed using *IBM SPSS Statistics* version 28 (IBM Corp., Armonk, NY, USA). As expected, we observed a significant deviation from normal distribution for the primary outcome in both groups [Shapiro-Wilk test, W (152) = 0.95, *p* < 0.001 for DF, W(1060) = 0.91, *p* < 0.001 for TF]. This was also true for the secondary outcome [Shapiro-Wilk test, W (46) = 0.92, *p* < 0.01 for DF, W(647) = 0.56, *p* < 0.001 for TF]. Thus, group differences for the primary and secondary outcomes were investigated with Mann-Whitney U tests for two independent samples, applying a significance level of *p* < 0.05.

## Results

### Participants

In the intervention group (DF), a total of *n* = 152 participants were included, all 4th year medical students. Data collection took place during the curricular course “Emergency Medicine Internship.”

The control group (TF) encompassed a total of *n* = 1,060 participants, corresponding to an average of 118 participants per semester (range: 110–130 participants). The final allocation ratio DF/TF was thus 1/7.

The present study used regularly assessed data from the Medical Faculty. Since this data is usually not related to other demographical information, gender and age of the participants were not assessed at the time of data acquisition. As a representative estimate for both groups, we selected the basic demography of the student cohort encompassing our intervention group (DF) at the first day of their studies, which was October 1st,2016. As of this date, the cohort consisted of 303 students (64.4% female, 35.6% male), with a median age of 19 years (range 17–34 years). The time between the first day of studies and data collection was rather consistent in each of the cohorts (~3 years and 8 months), resulting in an estimated median age of 22–23 years in both the intervention and the control group at the time of data collection.

#### RH1

For DF, the median score was 16.5 [standard deviation (SD) = 2.30]; for TF, the median score was 18.0 (SD=1.75). Students in the DF group scored significantly lower than students in the TF group (Mann-Whitney U = 50186.0; standardized test statistic = −7.65, *p* < 0.001).

#### RH2

A total number of *n* = 693 participants rated the course (DF: *n* = 46; TF: *n* = 647). For DF, the median score was 3.0 (SD = 1.45); for TF, the median score was 1.0 (SD = 0.66). Students in the DF group rated the course significantly worse than students in the TF group (Mann-Whitney *U* = 5,352.5; standardized test statistic = −9.04, *p* < 0.001).

**Figure 1:**
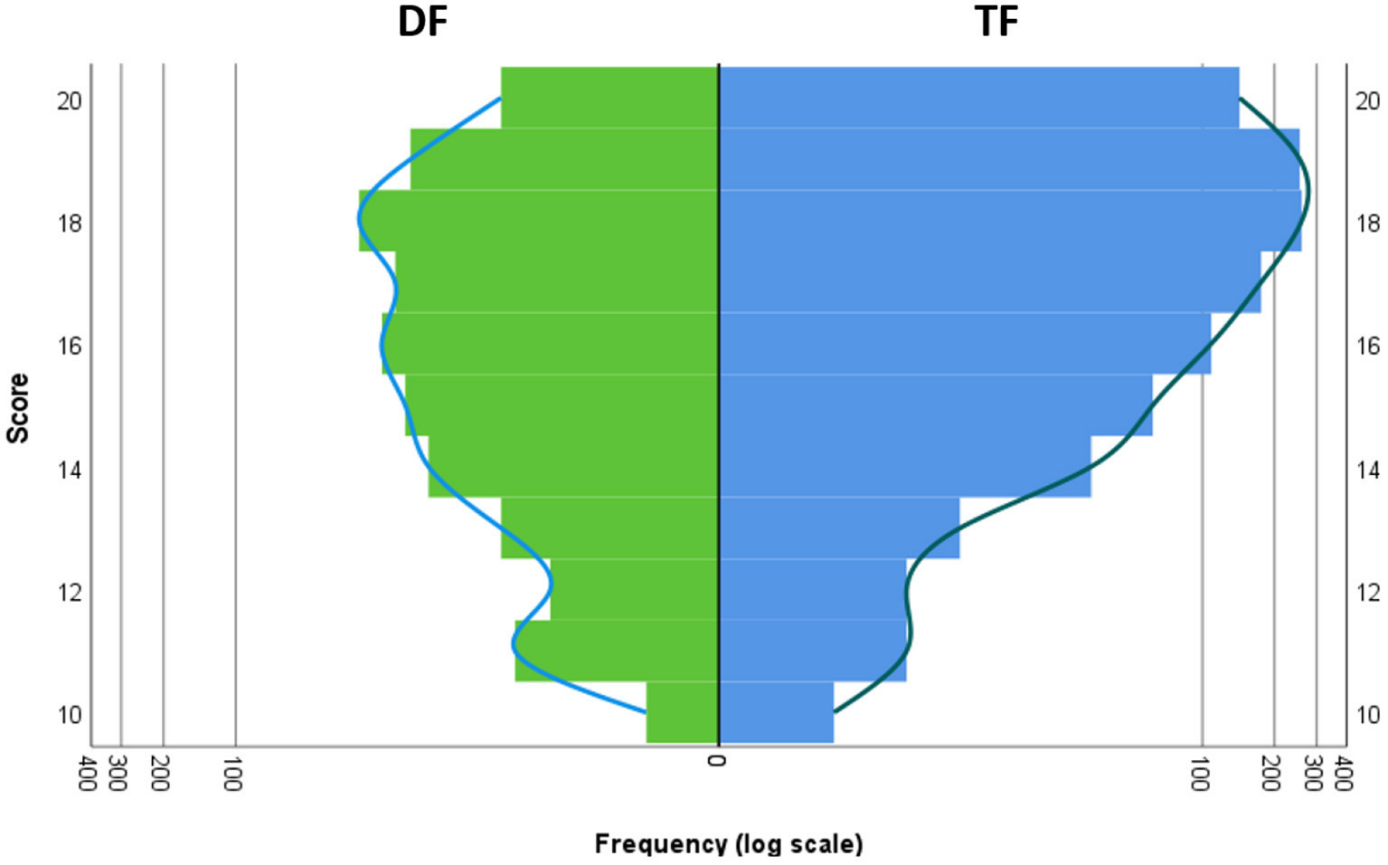
Comparison of DF and TF with respect to the frequency of the exam scores. Left/green: DF, right/blue: TF. The x axis shows the frequency of each score displayed on the y axis. To account for the different sample sizes of DF and TF, a logarithmic scale was used for the x axis.

**Figure 2:**
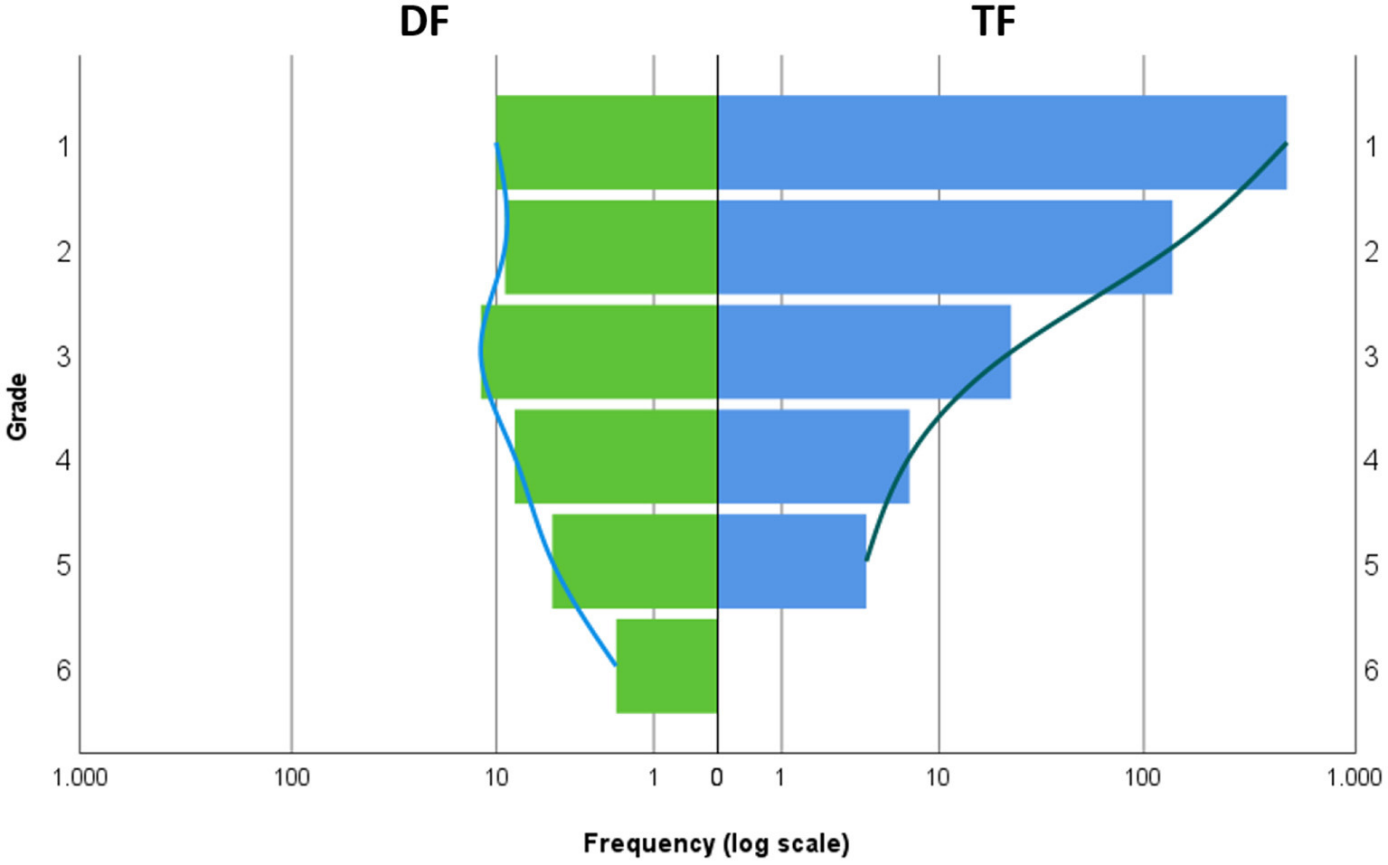
Comparison of DF and TF with respect to the frequency of school grades. Left/green: DF, right/blue: TF. The x axis shows the frequency of each grade displayed on the y axis. To account for the different sample sizes of DF and TF, a logarithmic scale was used for the x axis.

Figures 1, 2 compare the digital format (DF) and traditional format (TF) with respect to the exam score and school grade.

Moreover, we calculated effect sizes for RH1 and RH2. Following the methods described by Fritz et al. (26), we decided to use Pearson's r for both hypotheses, which is defined as $r=\frac{z}{\sqrt{N}}$. Effect size for the score in the final examination was *r* = 0.22; effect size of the school grade was *r* = 0.34. According to Cohen (27), these were small and medium sized effects, respectively.

To test for between-cohort variation in the historical control group, a one-factorial Kruskal-Wallis ANOVA of ranks was calculated for the 9 cohorts of TF. The result was significant [H(8)=207.98, *p* < 0.001], indicating a substantial variability in scores across semesters. For a visualization of the cohorts, please see Supplementary Figure 1.

Similarly, to the score variable, a one-factorial Kruskal-Wallis ANOVA of ranks was calculated for the 9 cohorts of TF for the school grade. The result was significant [H(8) = 16.67, *p* < 0.05], indicating again a substantial variability in scores across cohorts. For a visualization of the cohorts, please see Supplementary Figure 2.

The results illustrate that the findings within the historical control vary substantially, both for the score and for the school grade. We consider this a strong argument for including all available data sets in the control group of the present study. Thus, including all 9 cohorts improves not only statistical power, but also ecological validity of the study results.

The findings showed significant differences between the groups for both outcome variables. To rule out that these differences were caused by effects of novelty resp. non-familiarity of the teachers with DF, we incorporated data for the two subsequent semesters (winter term 2020/2021 and summer term 2021), which took place in a similarly newly developed hybrid format (HF) combining online and on-site elements. In HF, the hands-on training was resumed under strict hygienic measures (e.g., close monitoring of the participant's vaccinated, recovered or tested status, wearing face masks etc.), whereas theoretical lessons were still kept online. We compared HF with both TF and DF with respect to score and school grade. Remarkably, HF led to an intermediate performance: Scores were still lower than in the TF group (median of HF = 17.0, SD = 2.19; Mann-Whitney *U* = 1,014,047.5; standardized test statistic = −6.37, *p* < 0.001), but higher than in the DF group (Mann-Whitney *U* = 22,097.5; standardized test statistic = −2.39, *p* < 0.05). Concerning the school grade, HF was significantly better than DF (median of HF = 1.0, SD = 0.75; Mann-Whitney U = 1012.0; standardized test statistic = 6.67, *p* < 0.001) and on a comparable level to TF (Mann-Whitney U = 38364.0; standardized test statistic = −1.08, *p* = 0.28, n.s.). Looking at the two HF semesters in detail, we found that this improvement was already present from the very beginning, i.e. the winter term of 2020/21, where the median score (18.0) was even better than in the following summer term of 2021 (17.0). Moreover, HF achieved a median school grade of 1.0 from the first semester on.

## Discussion

The study results impressively illustrate that purely digital education for teaching clinical skills (5, 6) (5) (5) leads to worse performance in final exams compared to competency-based on-site learning in the field of emergency medicine. Findings from two subsequent semesters with a similarly new hybrid concept (HF) demonstrated that this effect can – at least to a substantial part - be attributed to the aspect of the teaching format.

This finding is particularly remarkable given the fact that the content and teaching form of the theoretical units were basically identical between the study arms (DF, TF). Thus, it is reasonable to assume that the essential difference between study arms was in the practical units—and still we found a substantial impact on the theory aspect. In terms of the spiral curriculum, this strongly argues for a mutual support of theory and practice in terms of learning success. In other words, theoretical knowledge benefits from practical application in simulation-based learning settings (18). Moreover, the students' subjective evaluation of the digital teaching format was significantly worse than for the on-site format. These findings point at some essential limitations in the application of digital education.

Incorporating data from the subsequent HF group demonstrates that the differences between study groups can indeed be attributed to the formats themselves and not to effects of novelty or familiarity. Novelty was also given for the hybrid format which was introduced in the winter term of 2020/21. Nonetheless, both the score and the school grade improved substantially, and this was the case from the very beginning on. Still, we cannot exclude that novelty and familiarity with the teaching formats contributed to the results; however, the present data clearly indicate an influence of the teaching format itself beyond this explanation.

A possible explanation for the higher effectiveness of the spiral curriculum with hands-on practical training may lie in Craik and Lockhart's “level of processing” model (28, 29). This model highlights the particular importance of the learning process for the later recall of the learning content (28). The ability to recall a learning content is a function of the depth of mental processing: Deeper processing of an information (e.g., understanding the information by experience and analysis) leads to a better memory recall than shallower processing (e.g., learning facts by heart). The levels of processing model has been confirmed in various studies and has become one of the most influential frameworks in cognitive psychology (30), and it has immediate implications for the findings of the present study. A high processing depth achieved, for example, trough practical experience and active application thus seems to favor encoding in long-term memory and better learning performance (28). This notion is also corroborated by recent findings on simulation-based learning (18–23).

In addition, the students' subjective evaluation of the course was significantly worse for the digital teaching format (DF) than for the traditional format (TF) or the hybrid format (HF) with hands-on teaching elements. This subjective evaluation of the digital format (lower grade) may also have an unfavorable effect on learning performance, e.g. by leading to lower learning motivation. A contributing aspect could be the “zoom fatigue” aspect: Zoom conferences are very tiring and exhausting. This is due to the following reasons, among others: excessive close-up viewing, cognitive load, increased self-assessment by staring at videos of oneself, and limited mobility (31). These factors may have led to a decline in attention and, consequently, to poorer knowledge acquisition. This finding would also be in line with the findings from the HF condition, which combined online and hands-on elements and, consequently, led to exam results that were intermediate between DF and TF. However, it should be noted that the subjective evaluation of HF was on a similar level as the one for TF; thus, it is reasonable to assume that the negative evaluation of DF cannot be attributed to digital settings per se, but instead seems to reflect certain aspects of digital teaching – in our case the practical application.

Furthermore, there are other potential reasons for the poor evaluation by the students. A recently published study shows that students are more likely to be dissatisfied with online learning and rate it significantly lower than faculty (32). One reason for this could be the lack of exchange or sharing and group learning in the digital format. Particularly in emergency medicine, group sizes of 6 are aimed for in order to train BLS (33–35). Many studies demonstrate the success of team-based learning (TBL) concepts in healthcare professions education (36–38). Another reason for the poor evaluation by the students could be the lack of communication skills in the digital setting (39). The loss of the constrained teaching of practical skills, communication skills, and team skills in digital learning formats is a very significant limitation.

An interesting observation is a difference in skewness of the distributions between the conditions. The most likely explanation for the latter is a ceiling effect in the TF group, which usually leads to a skewed distribution (cf. Figure 1), since the maximum value is fixed by definition. In other words, the skewed distribution is most likely a result of the high allover score level in TF. If the score level is lower, the distribution will be more symmetric (as in DF). The same effect appears to be true for the school grade as well.

This statistic effect may also be responsible for the more heterogeneous student evaluation of DF, which was in our case reflected by a larger SD and range compared to TF. Thus, the distribution is most likely a consequence of the generally lower ratings in DF. This is in line with findings from a recent review by Naciri et al. (40) who found that acceptance of digital teaching formats is generally only moderate among health profession students. The contributing factors are manifold and encompass usability and ease of use platforms, lecturer characteristics, system quality, the information provided, and available technical support (41).

Another noteworthy aspect is that the return rates of the evaluation were considerably different between the study groups: the mean return rate of TF was 60.90%, whereas the mean return rate of DF was only 30.26%. We can only speculate about the reasons for this difference; in our view, the most likely explanation is that the students in DF were oversaturated with digital content and thus not motivated to participate in another online activity. The essential question, however, is whether this had an influence on our study results. Differences in response rates can indeed be critical, e.g. if only those participants evaluated that were particularly unsatisfied with the course. However, looking at the statistics, we are confident that the latter was not the case in our study. For each of the three worst grades (4–6), even the absolute numbers in DF were higher than those in the entire TF group. Given the fact that DF was only one out of 10 semesters in total, it seems very unlikely that this finding was caused by a response bias. Specifically, the grades 4–6 made up for a total of 1.7% of the TF group evaluations- but for 32.6% of the DF group evaluations. Thus, we conclude that the evaluation differences reflect true dissatisfaction with DF and not a response bias.

There is theoretical content that can be taught very well digitally. However, as soon as complex clinical activities such as resuscitation are involved, practical instruction (within a learner's team) is indispensable in order to achieve the necessary depth of processing, as our results show.

These findings show the importance of further scientific review of different teaching concepts and their consequence for medical education. Specifically, if the decision is made again to discontinue hands-on practical teaching for whatever reason, we must be aware that a lack of essential knowledge may be the consequence. We need to ensure that future doctors are adequately trained and can start their careers without deficits. The results appear to be highly transferable to other educational settings where healthcare providers are prepared for their clinical work.

### Limitations

The present study used a non-randomized study design. In other words, the allocation of the participants/ students to the study arms could not be randomized due to the pandemic situation. A proper randomization would have been desirable but was not feasible. Instead, we used a design with a historical control. This is clearly a limitation; to minimize any possible bias arising from this fact, we undertook all efforts to keep as many variables as possible constant between study arms, both concerning the teaching formats and the student samples.

Another limitation is the discrepancy of ~10% in the number of hours of the two formats. This resulted not from differences in content but was due to the digital nature in the DF. Here, no room changes were needed, and no time was lost due to long pauses and scenario preparations.

A further limitation is that the present data set encompasses only one emergency medicine seminar. However, the sample size was considerable, and the results were remarkably clear; nonetheless, we need more data from other fields of medicine to establish the findings.

## Conclusion

These study results show how essential and indispensable practical hands-on teaching is in medical education for knowledge acquisition. The interlocking of theoretical knowledge and practical skills as ensured by the learning spiral is indispensable. Thus, Bloom's and Miller's paradigms are transferable to today's digital world. Moreover, it can be assumed that these results provide evidence that a pandemic-induced decision back to purely digital courses may substantially impair essential knowledge of future physicians.

## Data availability statement

The data analyzed in this study is subject to the following licenses/restrictions: Anonymized original data are available from the authors upon reasonable request. Requests to access these datasets should be directed to lvogt@ukaachen.de.

## Ethics statement

The studies involving human participants were reviewed and approved by Ethics Committee of the Faculty of Medicine, RWTH Aachen University, Aachen, Germany. Written informed consent for participation was not required for this study in accordance with the national legislation and the institutional requirements.

## Author contributions

LV contributed substantially and has written the manuscript. She is first author of this manuscript and thus all other authors agreed to her first authorship. MK performed statistical analysis and data support. HS and MS collected the data and stored them carefully. SS and MK contributed substantially to the data interpretation and to the content of the manuscript. SS, MK, and RR have critically revised the manuscript for important intellectual content. All authors have made contributions to the manuscript and reviewed and revised the manuscript. All authors contributed to the article and approved the submitted version.

## Funding

This trial was funded by the Department of Anaesthesiology, University Hospital RWTH Aachen, Medical Faculty, RWTH Aachen, Aachen, Germany.

## Conflict of interest

The authors declare that the research was conducted in the absence of any commercial or financial relationships that could be construed as a potential conflict of interest.

## Publisher's note

All claims expressed in this article are solely those of the authors and do not necessarily represent those of their affiliated organizations, or those of the publisher, the editors and the reviewers. Any product that may be evaluated in this article, or claim that may be made by its manufacturer, is not guaranteed or endorsed by the publisher.
