# Supplementary figures and images for: At the limits of digital education. The importance of practical education for clinical competencies learning in the field of emergency medicine: A controlled non-randomized interventional study
Source: Front Med (Lausanne). 2022 Sep 16;9:993337. doi: 10.3389/fmed.2022.993337 (PMC9523109; doi:10.3389/fmed.2022.993337)

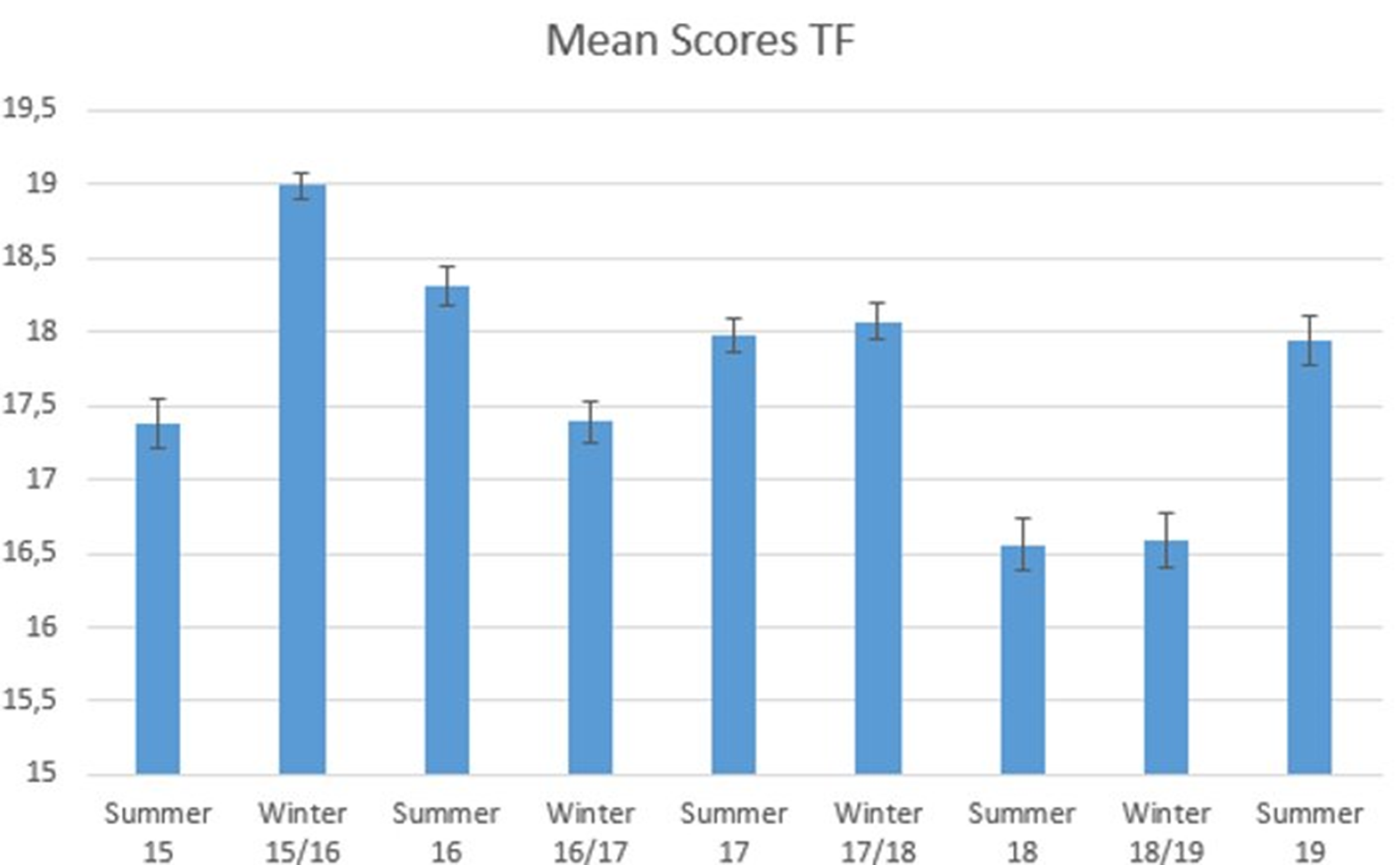

Supplement: Supplementary file 1 [file Image_1.tif]

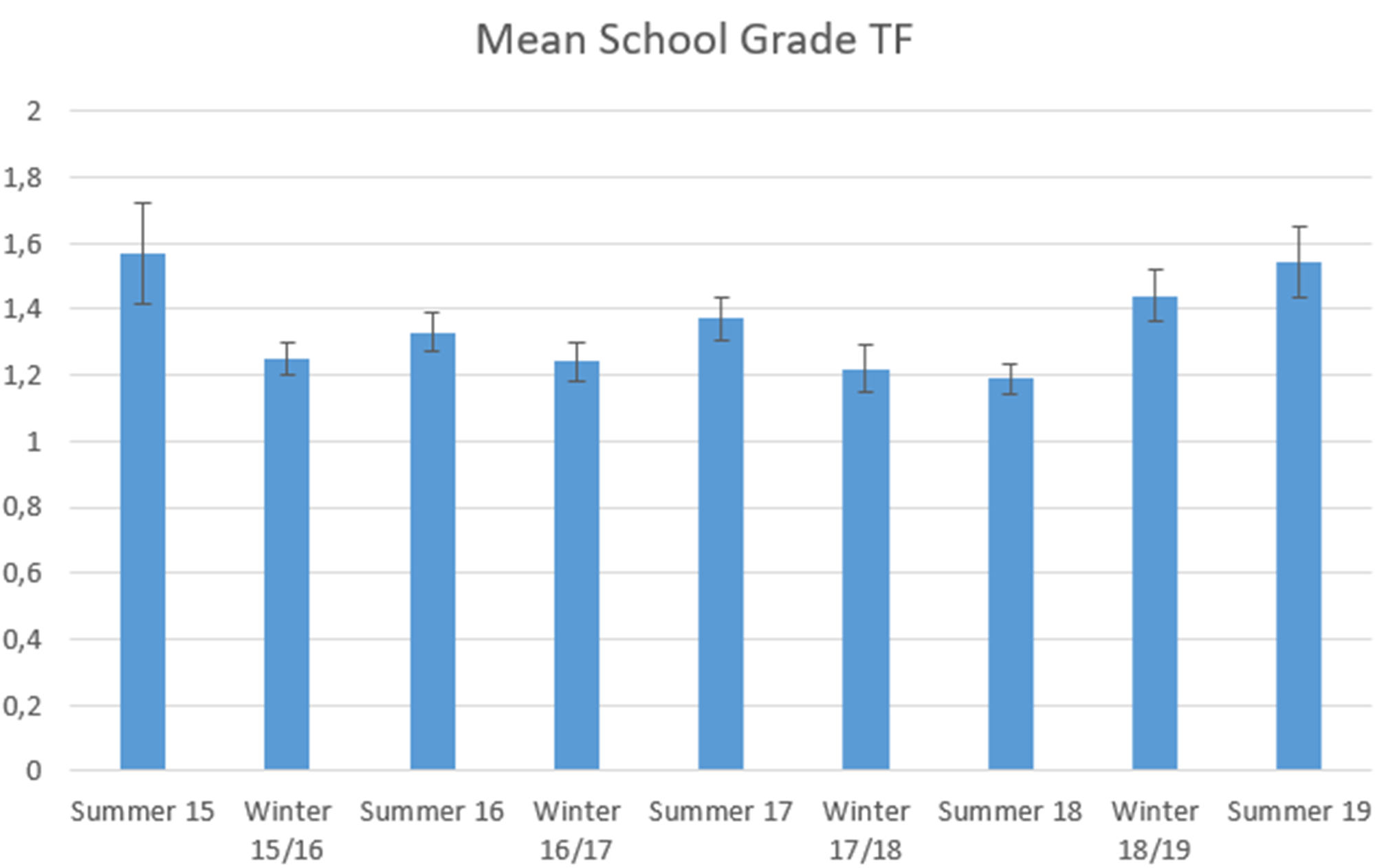

Supplement: Supplementary file 2 [file Image_2.tif]
